# Exploring the Effects of Coaching Behavior on the Mental Conditioning Process of Taekwondo Poomsae Athletes: A Moderated Mediation Effect of Psychological Needs and Gender

**DOI:** 10.3390/ijerph19127016

**Published:** 2022-06-08

**Authors:** Jaeyoun Jeong, Yunsik Shim, Myoungjin Shin

**Affiliations:** 1 Department of Sports Science, Soonchunhyang University, Asan 31538, Korea; tkdfun@daum.net; 2 Department of Leisure Sports, Kangwon National University, Samcheok 25913, Korea

**Keywords:** mental conditioning, multi mediated moderation, female, male, performance

## Abstract

The purpose of this study was to investigate whether there is a gender-specific difference in the indirect effect of perceived coaching behaviors of Taekwondo Poomsae athletes on performance mediated by basic psychological needs. To this end, a survey was conducted to collect data from 474 Taekwondo Poomsae athletes (male = 285). Based on the collected data, analysis of basic descriptive statistics and confirmatory factor analysis were performed, and PROCESS was used to test the proposed model of multi mediated moderation. For female athletes, there was an indirect effect of controlling coaching behaviors resulting in performance improvement mediated by autonomy, but this effect was not observed in male athletes. The findings of this study indicate that different mental conditioning strategies should be applied in consideration of athletes’ gender to improve coaches’ methods of instruction and ultimately improve performance in Taekwondo Poomsae.

## 1. Introduction

Athletes at the elite or professional level have a stronger desire to win than those at the recreational level. Coaching behaviors have a direct impact on the psychological and emotional responses of athletes and are some of the most important factors in predicting positive sports performance [1,2,3]. Coaching behaviors also have a direct impact on athletes’ emotions in training and competition, motivation for participation in sports, and satisfaction with their performance [4,5].

A mini-theory of the self-determination theory is the basic psychological needs theory which proposes that three basic psychological needs (BPN), namely, autonomy, competence, and relatedness, must be satisfied to foster well-being and health [6]. If a need is satisfied, it is highly correlated with positive variables such as subjective well-being, life satisfaction, positive emotion, and prosocial behavior [7], whereas when a need is unsatisfied, it leads to frustration and depression, and is highly correlated with problem behaviors such as anxiety and aggression [8].

Moreover, the satisfaction of athletes’ psychological needs is determined by coaching behavior [9,10,11]. For example, autonomy-supportive coaching behaviors enable athletes to choose from options within training standards and develop intrinsic motivation, understand the relevance of given tasks, and receive positive feedback from coaches, thus fulfilling their psychological needs. In contrast, controlling coaching behaviors operate only through negative conditions, rewards, and intimidation, and they focus on the performance outcome rather than the process, often resulting in frustration or interference with athletes’ psychological needs [12,13,14,15].

In educational literature, BPN satisfaction has been reported to have a positive effect on students’ academic achievement [16,17,18]. According to self-system process theory [19], when a student feels that the school fulfills her/his BPN well, she/he will be more likely to engage in school activities, in turn resulting in higher academic achievement. Although BPN have been shown to improve individual performance, few studies have empirically investigated the relationship between BPN and performance in the context of sports.

As sports are typically competitive, it would be inappropriate to assume that controlling coaching behaviors always have a negative impact on athletes’ performance. Amorose and Anderson-Butcher [20] investigated the effect of coaching behaviors on athletes’ motivation in a variety of individual and team sports, and the results showed that controlling coaching behaviors increased extrinsic motivation and lowered intrinsic motivation more than autonomy-supportive coaching behaviors. Thus, controlling coaching behaviors can be a driving force for the athletes to achieve a goal by enhancing the extrinsic motivation of the athlete.

Taekwondo is a sport that emphasizes ethical rules and an attitude of respect for tradition and other human beings, and it is based on a culture that respects the teacher’s authority [21]. Thus, Taekwondo can be considered a sport type in which the autonomy of individual players is more likely to be suppressed than respected. In Taekwondo it is likely that controlling coaching behaviors would promote performance but not fulfill the athletes’ BPN.

In general, conditioning in the sports domain includes physical conditioning to bring out the best performance in the athletes [22]. However, mental conditioning has also been shown to have a positive impact on performance [23]. To apply the effect of mental conditioning in a sports settings, the conditioning program must consider the individual characteristics of each athlete, including gender. Previous studies have reported a gender-specific moderating effect of BPN satisfaction on various predictors [24,25]. An effect of gender was also observed in the relationship between coaching behaviors and the BPN of athletes [26]. For the current study, we developed a model to predict how gender influences the effect of coaching behaviors on the process of mental conditioning that affects performance mediated by BPN. The specific research hypotheses are as follows:

**Hypothesis** **1** **(H1).** *Athlete’s gender will influence the indirect effect of autonomy* *-supportive coaching behaviors on performance mediated by BPN.*

**Hypothesis** **2** **(H2).** *Athletes’ gender will influence the indirect effect of controlling coaching behaviors on performance mediated by BPN.*

## 2. Materials and Methods

### 2.1. Participants

The participants of this study comprised 500 Taekwondo Poomsae athletes in middle school, high school, and university. Data from 474 participants (male athletes = 285) were used in the study, as 26 responses were excluded due to being incomplete. The mean age of the participants was 19.26 years (SD = 2.30), the mean duration of the training experience was 9.22 years (SD = 3.67), and the mean duration of the athletic career was 4.41 years (SD = 2.77).

### 2.2. Measures

#### 2.2.1. Autonomy-Supportive Coaching Behaviors

To assess autonomy-supportive coaching behaviors, we used six items from the Korean version of the Sport Climate Questionnaire by Amorose and Anderson-Butcher [27]. Each item is measured on a 7-point Likert scale (1 = strongly disagree, 7 = strongly agree). The Cronbach’s α of the scale was 0.947 in the current study, and via confirmatory factor analysis (CFA), $\chi^{2}$(9) = 176.90 (*p* = 0.000) we found the following values: Tucker-Lewis index (TLI) = 0.992, comparative fit index (CFI) = 0.997, and root means square error of approximation (RMSEA) = 0.058, indicating a good model fit. Good fit thresholds for these indices were CFI and TLI > 0.90, and RMSEA < 0.08 [28].

#### 2.2.2. Controlling Coach Behaviors

Controlling coach behaviors were measured using the Korean version of the Controlling Coach Behaviors Scale (CCBS) developed by Bartholomew et al. [14]. The CCBS includes 4 factors comprising 19 items: controlling use of reward (5 items), conditional regard (5 items), intimidation (5 items), and excessive personal control (4 items). Each item is measured on a 7-point Likert scale (1 = strongly disagree, 7 = strongly agree).

The results of the first-order CFA showed $\chi^{2}\left(146\right)=1$666.01 (*p* = 0.000), and the model fit did not meet the criteria (TLI = 0.767, CFI = 0.801, RMSEA = 0.124). Thus, a second-order CFA was performed. Four items with factor loading ≤0.40 (one item for each factor) were removed, and a second-order CFA was performed with the remaining 15 items. The null hypothesis was rejected as $\chi^{2}\left(84\right)=3$26.51 (*p* = 0.000), but the model fit indices met the criteria (TLI = 0.947, CFI = 0.958, RMSEA = 0.078). The Cronbach’s α was 0.874 for controlling use of reward, 0.928 for intimidation, 0.835 for excessive personal control, and 0.925 for negative conditional regard.

#### 2.2.3. BPN

To measure athletes’ BPN, we used a modified and supplemented version of the questionnaire used in Vallerand, Fortier, and Guay [29]. The modified questionnaire consisted of 3 factors comprising 9 items: autonomy (3 items), competence (4 items), and relatedness (2 items). Each item was measured on a 5-point Likert scale (1 = strongly disagree, 5 = strongly agree). The CFA indicated that the null hypothesis was rejected as $\chi^{2}\left(24\right)=4$2.52 (*p* = 0.000), but other fit indices indicated a good model fit of the data (TLI = 0.989, CFI = 0.993, RMSEA = 0.046). The Cronbach’s α was 0.905 for autonomy, 0.941 for relatedness, and 0.934 for competence.

#### 2.2.4. Performance

The performance of the participants was calculated as whether they had placed 3rd or higher in national competitions within the last year (3 = yes, 1 = no) multiplied by the number of times the participant had placed 3rd or higher in those competitions (1 = 1 time, 2 = two times, 3 = 3 or more times. The range of the performance value was from 1 point to 9 pints.

### 2.3. Procedures

The study survey was conducted in the off-season without any competitions. Prior to the survey, we communicated with the coach of each team to introduce the researcher, inform them of the intention and purpose of this study, and obtain permission to conduct the survey. Google Forms was used to collect data for the survey. The coaches were asked to explain the purpose and content of the study to their athletes, and the survey was conducted only after the athletes expressed their written approval to voluntarily participate in the study.

### 2.4. Analysis

**Figure 1:**
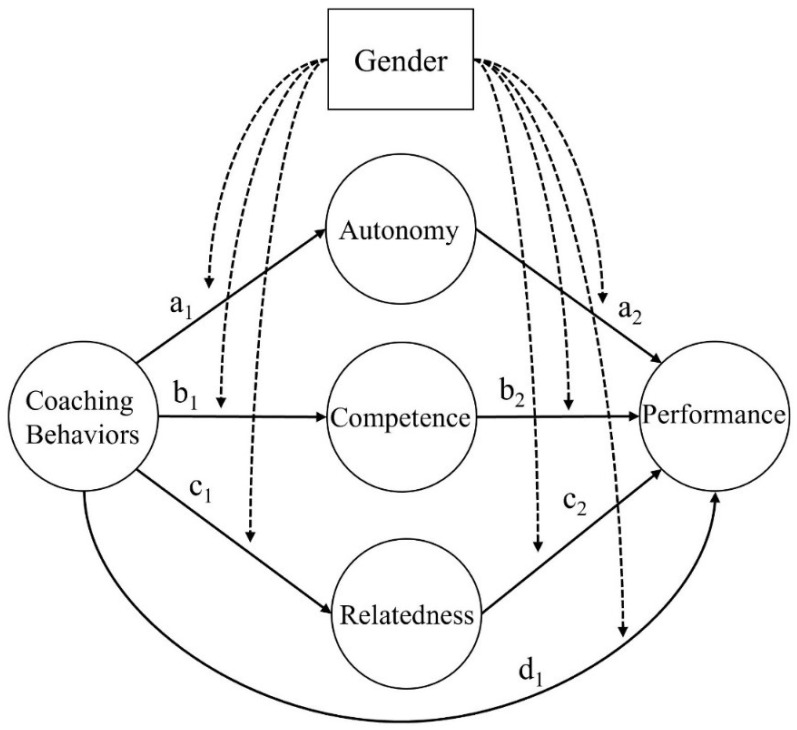
The proposed model in this study.

Using SPSS ver. 23, basic descriptive analysis and reliability analysis were performed, and CFA was tested using AMOS v23. The moderated mediation model (Model 59) in PROCESS [30] was used to test the multi-mediated moderation model in Figure 1. All input variables were converted into standardized scores for the analysis. The statistical significance level was set to 0.05.

## 3. Results

### 3.1. Descriptive Statistics and Correlation Analysis

The correlation analysis between the variables showed that autonomy-supportive coaching behaviors had a negative correlation with factors of the controlling coaching behavior (−0.34 to −0.52) but a positive correlation with BPN (0.13 to 0.44). A negative correlation (−0.04 to −0.39) was observed between BPN and factors controlling coaching behavior, and since there was no case of high correlation between variables, the likelihood of multicollinearity was expected to be low. The kurtosis and skewness of all variables were ≤±2, showing a normal distribution (Table 1).

### 3.2. The Gender-Specific Effect of Controlling Use of Reward on Performance Mediated by BPN

The age, athletic career, and training experience were input as covariate variables, and we examined whether there was a gender-specific difference in the effect of controlling use of reward on performance mediated by BPN. As shown in Table 2, controlling use of reward had a significant effect only on autonomy (β = −0.24, t = 2.68, *p* < 0.01). For autonomy, the standardized regression coefficient on performance was −0.17 (t = 2.02, *p* < 0.05), and there was a marginal gender-specific moderating effect (β = 0.16, t = 1.96, *p* = 0.051). Using bootstrapping, the difference in indirect effect on CRU → Autonomy (MV1) → Performance (DV) depending on the gender of the athletes was tested. The indirect effect was 0.02 for female athletes, and the result was statistically significant because 0 was not included in the 95% confidence interval. The indirect effect among male athletes was not significant. Therefore, unlike male athletes, female Poomsae athletes experience performance improvement mediated by autonomy when exposed to the coaching environment of controlling use of reward.

### 3.3. The Gender-Specific Effect of Conditional Regard(CR) on Performance Mediated by BPN

The age, athletic career, and training experience were input as covariate variables, and it was examined whether there was a gender-specific difference in the effect of conditional regard on performance mediated by BPN. As shown in Table 3, the effect of conditional regard on autonomy (β  = −0.36, t = 4.69, *p* < 0.001) and relatedness (β  = −0.28, t = 3.50, *p* < 0.001) were significant. The standardized regression coefficient of autonomy on performance was −0.18 (t = 2.19, *p* < 0.05), and there was a marginal gender-specific moderating effect (β  = 0.16, t = 1.86, *p* = 0.062). Using bootstrapping, the difference in indirect effect on conditional regard → Autonomy (MV1) → Performance (DV) depending on the gender of the athletes was tested. The indirect effect among female athletes was 0.07, and the result was significant because 0 was not included in the 95% confidence interval. The indirect effect among male athletes was not statistically significant. Therefore, unlike male athletes, female Poomsae athletes experience performance improvement mediated by autonomy when exposed to the coaching environment of conditional regard. However, the multi-mediated moderation effect illustrated in Figure 1 was not observed with autonomy-supportive coaching behaviors, intimidation, or excessive personal control.

## 4. Discussion

In the present study, it was found that controlling coaching behaviors in sports may have a negative effect on BPN satisfaction but a positive effect on performance. Unlike the general educational environment [16,17,18], athletes in sports aim for peak performance in competition settings, thus the coaching environment is coercive and controlling. Thus, in terms of improving the performance of athletes, such a controlling coaching environment is beneficial. Since the participants of this study are Asian Taekwondo athletes, which emphasizes a disciplinarian and hierarchical culture, we cannot rule out the possibility that controlling coaching behaviors may lead to performance improvement of the athletes. As the presence of cultural differences in coaching styles was reported in a previous study [31], further studies should be conducted by expanding the scope to a socio-cultural comparative study including Western athletes and different sport types.

When the signs of the direct effect and the indirect effect are the opposite there may be a suppression effect [32]. In this study, the sign of the indirect effect was positive on the path of conditional regard → autonomy → performance, whereas the sign of the direct effect was negative, indicating the possibility of a suppression effect. However, in the correlation between performance and conditional regard was larger than the size of the direct effect, so the possibility of a suppression effect is low. Therefore, in this study, autonomy was a mediator variable in the relationship between controlling coaching behaviors and performance.

The results of this study show that there was an indirect effect of controlling coaching behaviors on performance mediated by autonomy among female athletes, which was not the case with male athletes. Therefore, if female Taekwondo Poomsae athletes are exposed to controlling coaching behaviors, a mental conditioning strategy that can enhance autonomy may have a positive effect on performance. For example, a recent study reported a positive correlation between goal clarity and autonomy [33], so it may be beneficial for athletes to select a clear goal based on their own decision to enhance autonomy and ultimately improve performance.

In general, controlling coaching behaviors have been reported to increase burnout in athletes [34,35], interfere with commitment to the sport [36], and increase athletes’ antisocial behavior [37]. In other words, controlling coaching behaviors can be viewed as having a negative impact on several predictors. However, the results of this study supported the idea that controlling coaching behaviors may have a positive effect on performance, and further studies are needed to continue the investigation.

Our findings confirmed a gender-specific difference in the effect of controlling coaching behaviors. This is supported by Koka and Sildala [38], who found a gender-specific difference in the effect of teachers’ controlling behaviors. Our findings may be due to female athletes being more adaptive to a controlling coaching environment compared to male athletes or their ability to better accept opinions and feedback from their coaches, leading to improved performance. Future studies should investigate the effect of gender on learning ability in a controlling environment. In this study, we confirmed the importance of strategies enhancing autonomy for female athletes, but effective mental conditioning strategies for male athletes could not be identified. Further research is required to investigate mental conditioning strategies for male athletes that can enhance their performance.

## 5. Conclusions

We investigated the mental conditioning process in which coaching behavior helps to improve the performance of Taekwondo athletes. Results showed that there was a negative association with the BPN of controlling coaching behavior, but a positive association with autonomy-supportive coaching behaviors. The indirect effect of controlling coaching behavior on performance through BPN differed according to gender. In female athletes, the controlling coaching behavior had a positive effect on performance through autonomy, whereas in male athletes, there was no significant indirect effect. This result can be seen as a mental conditioning strategy that has the potential to improve performance for female players by controlling coaching behavior.

Participants of different ages may respond differently to the coach’s authority and coaching style. A study by Partington, Cushion, and Harvey [39] found that coaches teaching younger participants used more instruction and those teaching older participants used more divergent questioning and significantly more total feedback and punitive behaviors. The coaches of the younger age groups used more training form activities than the coaches of the older age groups who used more playing form activities. In the current study, participants were middle school, high school, and university athletes, so bias by age is a possibility. However, as shown in Table 2 and Table 3, age was added with covariate to statistically control the age to supplement the limitations of the study. As the current study was conducted on Asian athletes, there are limitations in generalizing the study results, and the explanation of the study protocol might also add bias to how the participants would answer.

## Figures and Tables

**Table 1:** Descriptive statistics and correlation analysis.

	1	2	3	4	5	6	7	8	9
1. ASCB	1								
2. CUR	−0.34 **	1							
3. Intimidation	−0.52 **	0.50 **	1						
4. EPC	−0.47 **	0.43 **	0.72 **	1					
5. CR	−0.52 **	0.60 **	0.78 **	0.64 **	1				
6. Autonomy	0.44 **	−0.21 **	−0.38 **	−0.39 **	−0.38 **	1			
7. Competence	0.13 **	−0.13 **	−0.04	−0.06	−0.07	0.29 **	1		
8. Relatedness	0.30 **	−0.22 **	−0.25 **	−0.27 **	−0.31 **	0.40 **	0.28 **	1	
9. Performance	0.01	−0.04	−0.08	−0.05	−0.07	0.06	0.25 **	0.15 **	1
Mean	5.20	3.07	2.40	2.39	2.77	4.03	3.08	4.09	4.09
SD	1.31	1.38	1.36	1.27	1.38	0.84	0.99	0.87	3.56
Skewness	−0.53	0.01	0.76	0.79	0.59	−0.50	0.01	−0.78	0.22
Kurtosis	−0.14	−0.79	−0.17	0.04	−0.28	−0.56	−0.22	0.21	−1.47

ASCB = autonomy-supportive coaching behaviors; CUR = controlling use of reward; EPC = excessive personal control; CR = conditional regard. ** Correlation is significant at the 0.01 level (2-tailed).

**Table 2:** Regression coefficients and model summary information for multi mediated moderation (IV = CUR).

		Autonomy (MEV1)		Competence (MEV2)		Relatedness (MEV3)		Performance (DV)	
		Path	β	Path	β	Path	β	Path	β
IV	CUR ^a^	a_1_	−0.24 **	b_1_	0.05	c_1_	−0.13	d_1_	0.01
	Autonomy ^c^							a_2_	−0.17 *
	Competence ^d^							b_2_	0.20 *
	Relatedness ^e^							c_2_	0.06
MOV	Gender ^b^		0.12		0.26 ***		0.17 *		−0.38 ***
COV	Age		−0.10 *		−0.08		−0.08 ^†^		−0.13 **
	Training experience		0.08		0.06		0.11 *		−0.00
	Athletic career		0.05		0.35 ***		0.14 **		0.35 ***
IT	a × b		0.03		0.12 ^†^		−0.06		0.00
	c × b								0.16 ^†^
	d × b								−0.05
	e × b								0.01
*R* ^2^		0.06 ***		0.19 ***		0.10 ***		0.22 ***	
(∆*R*^2^)	a × b	0.00		0.00 ^†^		0.00		0.00	
	c × b							0.01 ^†^	
	d × b							0.00	
	e × b							0.00	

CUR = controlling use of reward; MEV = mediator variable; DV = dependent variable; IV = independent variable; COV = covariate; MOV = moderator variable; IT = interaction term. † *p* < 0.10, * *p* < 0.05, ** *p* < 0.01, *** *p* < 0.001.

**Table 3:** Regression coefficients and model summary information for multi mediated moderation (IV = CR).

		Autonomy (MEV1)		Competence (MEV2)		Relatedness (MEV3)		Performance (DV)	
		Path	β	Path	β	Path	β	Path	β
IV	CR ^a^	a_1_	−0.36 ***	b_1_	0.00	c_1_	−0.28 **	d_1_	−0.01
	Autonomy ^c^							a_2_	−0.18 *
	Competence ^d^							b_2_	0.22 **
	Relatedness ^e^							c_2_	0.07
MOV	Gender ^b^		0.04		0.29 ***		0.13 ^†^		−0.41 ***
COV	Age		−0.10 *		−0.07		−0.07		−0.13 **
	Training experience		0.05		0.05		0.08 ^†^		−0.01
	Athletic career		0.05		0.37 ***		0.15 **		0.35 ***
IT	a × b		−0.01		−0.02		−0.00		−0.05
	c × b								0.16 ^†^
	d × b								−0.06
	e × b								−0.00
*R* ^2^		0.15 ***		0.15 ***		0.14 ***		0.22 ***	
(∆*R*^2^)	a × b	0.00		0.00		0.00		0.00	
	c × b							0.01 ^†^	
	d × b							0.00	
	e × b							0.00	

CR = conditional regard; MEV = mediator variable; DV = dependent variable; IV = independent variable; COV = covariate; MOV = moderator variable; IT = interaction term. † *p* < 0.10, * *p* < 0.05, ** *p* < 0.01, *** *p* < 0.001.

## Data Availability

Data generated and analyzed during this study are included in this article. Additional data are available from the corresponding author on request.
